# The impact of the Beirut blast on the COVID-19 situation in Lebanon

**DOI:** 10.1007/s10389-021-01562-6

**Published:** 2021-05-22

**Authors:** Mohamad Y. Fares, Umayya Musharrafieh, Abdul Rahman Bizri

**Affiliations:** 1 College of Medical, Veterinary, and Life Sciences, University of Glasgow, Glasgow, Scotland UK; 2 Faculty of Medicine, American University of Beirut Medical Center, Beirut, Lebanon; 3 Neuroscience Research Center, Faculty of Medical Sciences, Lebanese University, Beirut, Lebanon; 4 Department of Family Medicine, Director, COVID-19 Clinic, American University of Beirut Medical Center, Beirut, Lebanon; 5 COVID-19 Taskforce, Lebanese Society for Infectious Diseases and Clinical Microbiology, Beirut, Lebanon; 6 Division of Infectious Diseases, Department of Internal Medicine, American University of Beirut Medical Center, Beirut, Lebanon; 7 National Committee for Communicable Diseases, Beirut, Lebanon

**Keywords:** Beirut blast, Novel coronavirus disease 2019, COVID-19, Lebanon

## Abstract

**Aim:**

On August 4, 2020, a massive explosion hit Lebanon’s capital city, Beirut. The aim of this study was to explore the effect of the Beirut blast on the COVID-19 situation in the country.

**Subject and Methods:**

Data on COVID-19 were retrieved from the Lebanese Ministry of Public Health (LMOPH), where all the COVID-19 positive cases were reported. The study was divided into two periods, considering the incubation period of the COVID-19 virus: (July 27–August 9, 2020) and (August 10–23, 2020). Information obtained included daily number of cases, tests, deaths, hospitalized patients, intensive care unit (ICU) patients, and mode of acquisition (local vs. expat). Daily positivity rates were reported per 100 tests. An independent sample t-test and a Joinpoint regression analysis were used to determine significance. A *p* value less than 0.05 was considered significant.

**Results:**

A total of 201,010 tests were conducted during our studied period, with 8993 positive cases, constituting a total positivity rate of 4.5 per 100 tests. Case fatality rate over the studied period was 0.8%. The positivity rate of the period prior to August 10, 2020, was 2.7 per 100 tests, significantly less than that of the period following the explosion, which was 6.4 per 100 tests (*p* < 0.001). During our studied period, daily positivity rates were significantly increasing at a slope of 0.29 (*p* < 0.001). A significant increase in slope was noted on August 13, 2020 (*p* < 0.001). The number of hospitalized patients increased from 139 patients on July 27 to 266 on August 23, 2020, and that of ICU patients increased from 36 to 75.

**Conclusion:**

The port of Beirut explosion resulted in a significant increase in the daily number of positive COVID-19 cases. The aftermath of the explosion, the damage to healthcare facilities, and the overcrowding due to emergency efforts were contributing factors to that increase.

## Introduction

The first case of the novel coronavirus 2019 (COVID-19) in Lebanon was diagnosed on February 21, 2020 (MOPH 2020). At that time, the Lebanese people had many other ordeals to deal with, and the COVID-19 pandemic further exacerbated their challenging situation. Social and political turmoil had erupted earlier in the country, after popular uprisings emerged against the corruption and mismanagement of the political regime (Neal and Tansey 2020; Nuwayhid and Zurayk 2019). In addition, an economic crisis evident by the collapse of the banking system and a shortage of foreign currency led the country to bankruptcy, and its citizens to detrimental levels of poverty (Neal and Tansey 2020; Nuwayhid and Zurayk 2019). Concurrently, COVID-19 struck Lebanon and led to a multifaceted impact on the health, economic and educational sectors, among others (Bizri et al. 2020).

Initially, the Lebanese government took quick action to help limit the spread of the disease (MOPH 2020; Bizri et al. 2020). A nationwide lockdown took place, curfews were set, and learning institutions were temporarily closed (MOPH 2020; Bizri et al. 2020). While these non-pharmacologic measures were relatively successful in hindering the spread of the epidemic, their social and economic repercussions were detrimental and devastating (MOPH 2020; Bizri et al. 2020). The government was forced to start gradually easing off the restrictions and began reopening the country allowing expats to return, leading to a nationwide increase in the number of COVID-19 cases (MOPH 2020; Bizri et al. 2020).

On August 4, 2020, a massive explosion hit the capital city of Beirut, resulting in more than 190 deaths, 6000 injuries, 10–15 billion USD worth of property damage, and displacing 300,000 people from their homes (Devi 2020). The catastrophic blast was linked to approximately 2700 tons of confiscated ammonium nitrate that had been stored in the port without proper safety measures (Devi 2020). The explosion damaged many nearby hospitals, overcrowded healthcare facilities with patients, and reignited the popular uprising against the political regime (Devi 2020). Amid the already limited medical resources and supplies, the COVID-19 situation in the country had never been worse.

The aim of this study is to explore the epidemiological trends of the COVID-19 pandemic in Lebanon prior to, and after August 4, 2020, to assess the impact of the Beirut blast on the healthcare system of the country and its COVID-19 response.

## Methods

### Study design

In this descriptive analysis, we attempt to explore the catastrophic impact of the Beirut blast on the already debilitating COVID-19 burden in the Lebanese population. A relevant literature review pertaining to the topic was conducted. Daily COVID-19 reports were screened and analyzed up until August 23, 2020. The data were deidentified and publicly accessible from the LMOPH Epidemiologic Surveillance Unit website (MOPH 2020). Our database included the number of new cases, total cases, deaths, tests conducted, hospitalized patients, and status of cases (locals vs expats). COVID-19 is known to have approximately 4–7 days of incubation; hence, cases that occurred on August 4th would probably be detected around the 9th of August (Li et al. 2020). In addition, the Lebanese government imposed a lockdown that started in late August. Accordingly, in order to alienate confounding variables due to the lockdown imposed, and taking into account the incubation period of COVID-19, we chose to define our study period between July 27–August 23, 2020. We also divided the study period into two time intervals: July 27–August 9, 2020 (cases occurring prior to the Beirut blast), and August 10–23, 2020 (cases occurring due to the Beirut blast).

### Data analysis

In order to remove bias when comparing between the two time periods, we divided the number of daily cases by the number of daily tests conducted. Accordingly, positivity rates were reported per 100 tests. An independent sample t-test was used to check for any statistical significances between the mean incidence rates of the two periods. The Joinpoint regression analysis tool was then used to assess whether any significant changes occurred with respect to the trends of COVID-19 incidence during our studied period. For Joinpoint, we used a grid search model, with a maximum of 1 Joinpoint due to the relatively short time period under study. The model selection method was chosen to be permutation test, and the error options were set as to fit an uncorrelated errors model. A *p* value less than 0.05 was considered significant.

## Results

**Fig. 1:**
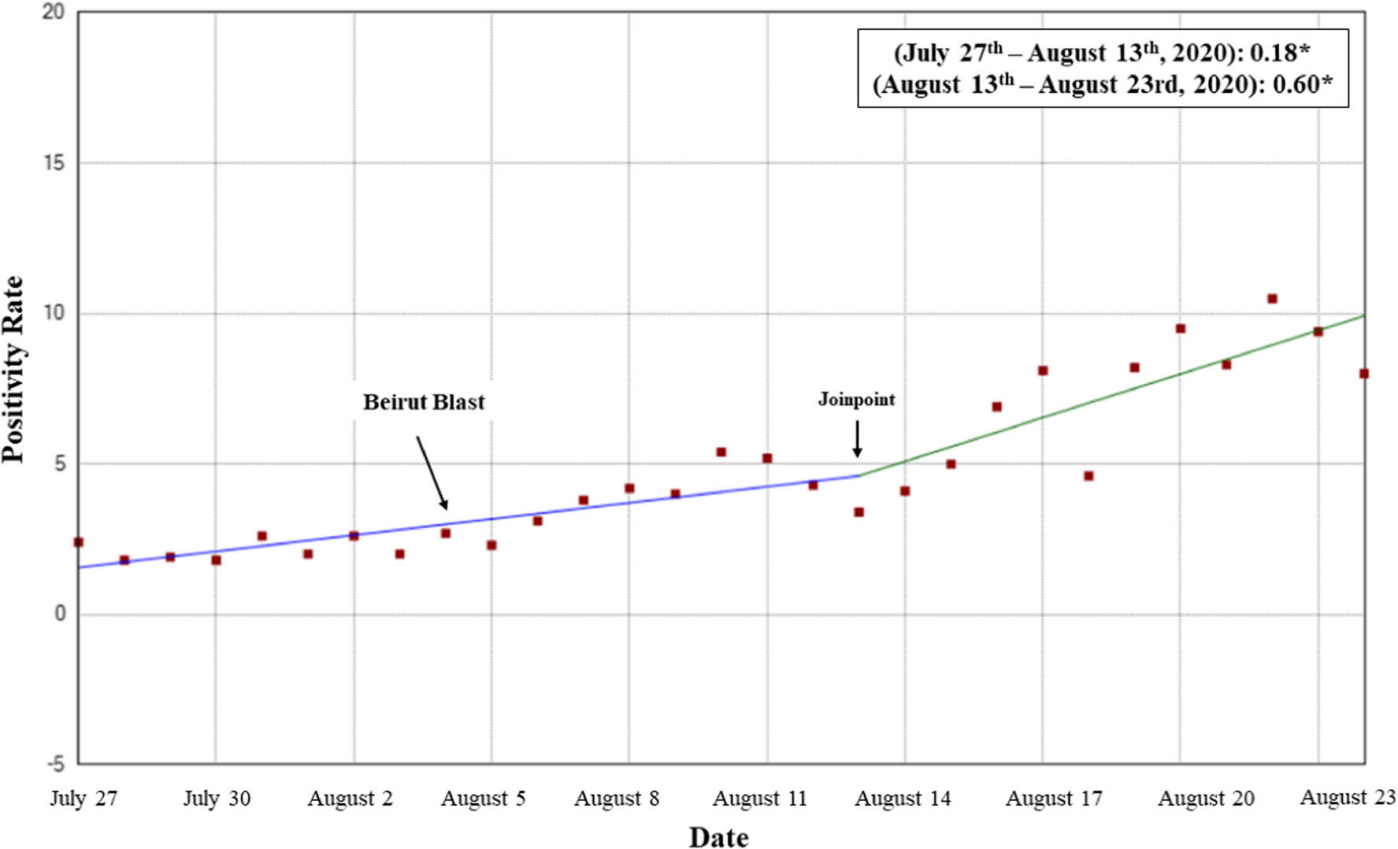
Trendline showing the daily COVID positive cases reported (per 100 tests) during our studied period (July 27–August 23, 2020)

**Fig. 2:**
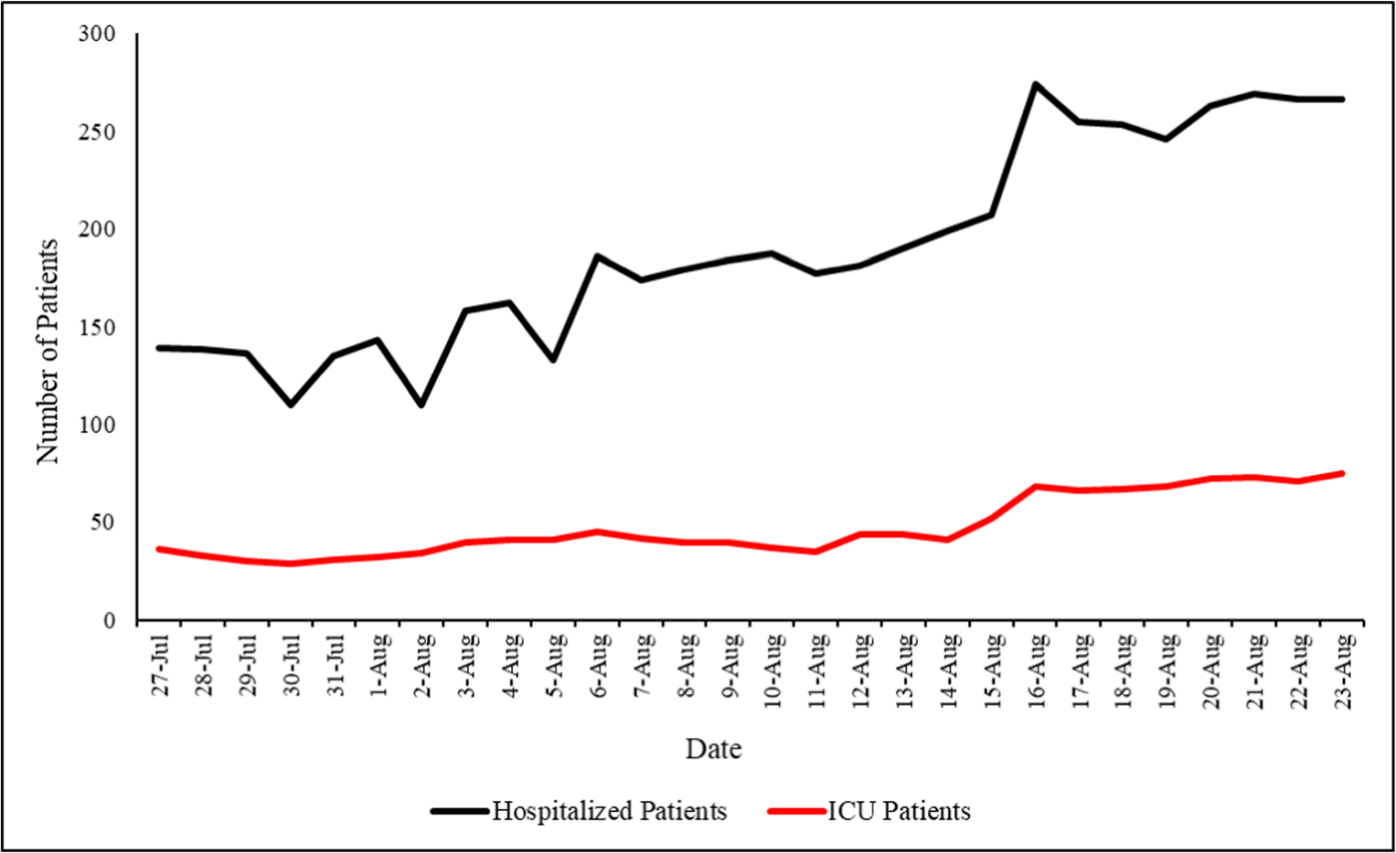
Trend showing the number of hospitalized patients and ICU patients during our studied period (July 27–August 23, 2020)

A total of 201,010 tests were conducted during our study period (July 27–August 23, 2020); out of which, 8993 cases had a positive result, constituting a positivity rate of 4.5 per 100 tests (Fig. 1). Out of the 8993 positive cases, 8652 (96%) were of local origin, while 341 (4%) were from expats entering the country. In addition, a total of 72 deaths occurred during our study period, constituting a case fatality rate of 0.8%. The number of hospitalized patients was 139 on July 27th, with 36 patients residing in the intensive care unit (ICU). This number rose to 266 patients on August 23rd, out of which 75 resided in the ICU (Fig. 2). The positivity rate over the entire studied period reported a significantly increasing slope of 0.29 (*p* < 0.001). When assessing for a significant change in slope during our studied period, a Joinpoint change on August 11, 2020 showed a significant increase from a slope of 0.18 (July 27–August 11, 2020) to 0.6 (August 12–23, 2020) (*p* < 0.001) (Fig. 1).

### Before the Beirut blast (July 27–August 9, 2020)

**Fig. 3:**
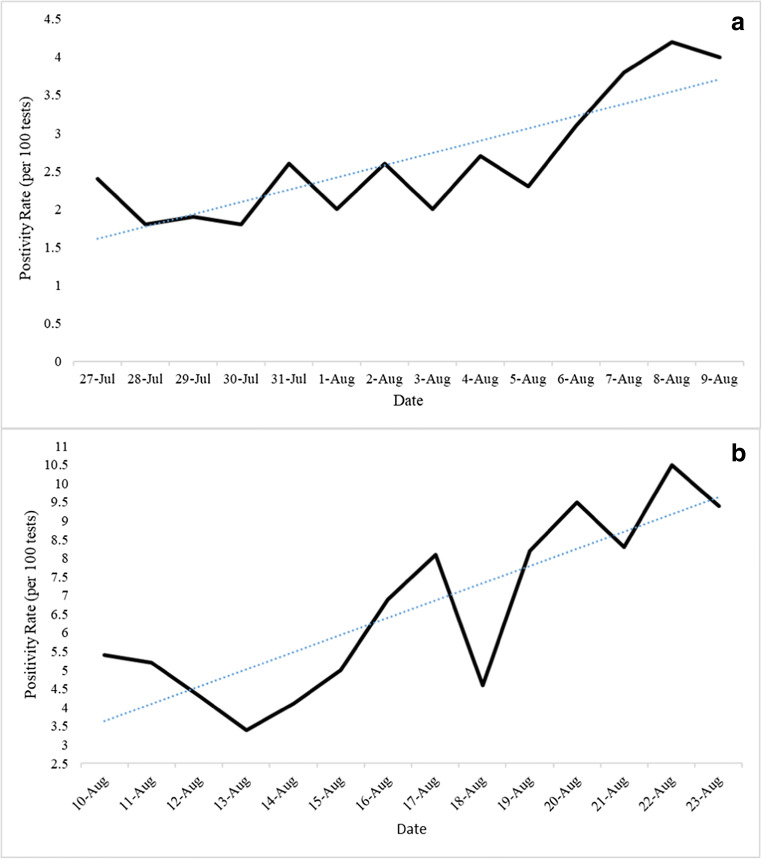
Trend of the daily COVID positivity rates (per 100 tests) in the time period prior to the Beirut blast **(a)** [slope = 0.16 per 100 tests], and the period following the Beirut blast **(b)** [slope = 0.46 per 100 tests]

A total of 104,210 tests (52%) were conducted in the month prior to the Beirut blast; out of which 2812 cases (31%) had a positive result, constituting a positivity rate of 2.7 per 100 tests. This rate was significantly lower than that reported in the month following the Beirut blast (*p* < 0.001; CI[−5.4,–2.6]). Out of the 2812 positive cases, 2637 (94%) were local cases, whereas 175 (6%) were expats. A total of 25 deaths occurred during this period, constituting a case fatality rate of 0.9%. As of August 9, 2020, there were 184 hospitalized patients, 40 of which were residing in the ICU (Fig. 2). The positivity rate of this period had a significantly increasing slope of 0.16 (*p* < 0.001) (Fig. 3a).

### After the Beirut blast (August 10–23, 2020)

A total of 96,800 tests (48%) were conducted in the month following the Beirut blast; out of which 6181 (69%) had a positive result, constituting a positivity rate of 6.4 per 100 tests. This rate was significantly higher than that reported in the month prior to the Beirut blast (*p* < 0.001; CI[−5.4,–2.6]). Out of the 6181 positive cases, 6015 (97%) were local cases, while 166 (3%) were expats. A total of 47 deaths occurred during this month, constituting a case fatality rate of 0.8%. As of August 23, 2020, there were 266 hospitalized patients, out of which 75 were residing in the ICU (Fig. 2). The positivity rate of this period had a significantly increasing slope of 0.46 (*p* < 0.001) (Fig. 3b).

## Discussion

The period following the Beirut blast showed a significantly higher daily COVID-19 positivity rates, number of deaths, and a higher increasing slope when compared to the period prior to the explosion. In addition, the number of hospitalized patients and those residing in the ICU increased by more than twofold. A point of significant change in slope was shown on the 13th of August. Considering the fact that COVID-19 has an incubation period of approximately 5 days, these findings emphasize the impact of the Beirut blast on the already struggling health sector in Lebanon, and the major setbacks that ensued regarding the COVID-19 situation, as a result.

### COVID-19 situation prior to Beirut blast

After the first wave of COVID-19 hit Lebanon in March 2020, the government quickly took decisive actions and strict measures to help limit the spread of the disease (Bizri et al. 2020). The crisis-stricken country initiated a total lockdown: the airport was closed for visitors and expats, restaurants and businesses were disrupted, and schools and universities adopted online-teaching protocols to help contain disease spread (Bizri et al. 2020; Azhari 2020). In addition, a public curfew was set, and masks were made mandatory in public spaces (Khamis 2019). This took a toll on the population’s socioeconomic status as many people lost their jobs, businesses had to shut down, and inflation was rising (Bizri et al. 2020; Aawast 2020). In addition, the country’s financial sector was suffering, as the airport closure exacerbated the already existing shortage of foreign currency (Bizri et al. 2020; Trading Economics 2020). Around late May, the government decided to reopen the country, after COVID-19 incidence reached minimal rates: the airport was reopened to visitors, restaurants and businesses were allowed to reopen with limited capacity, and the public curfew was lifted (Knecht 2020). Soon enough, COVID-19 cases started to increase progressively, but not at an alarming rate that would require significant measures (MOPH 2020).

### Consequences of the Beirut blast

**Fig. 4:**
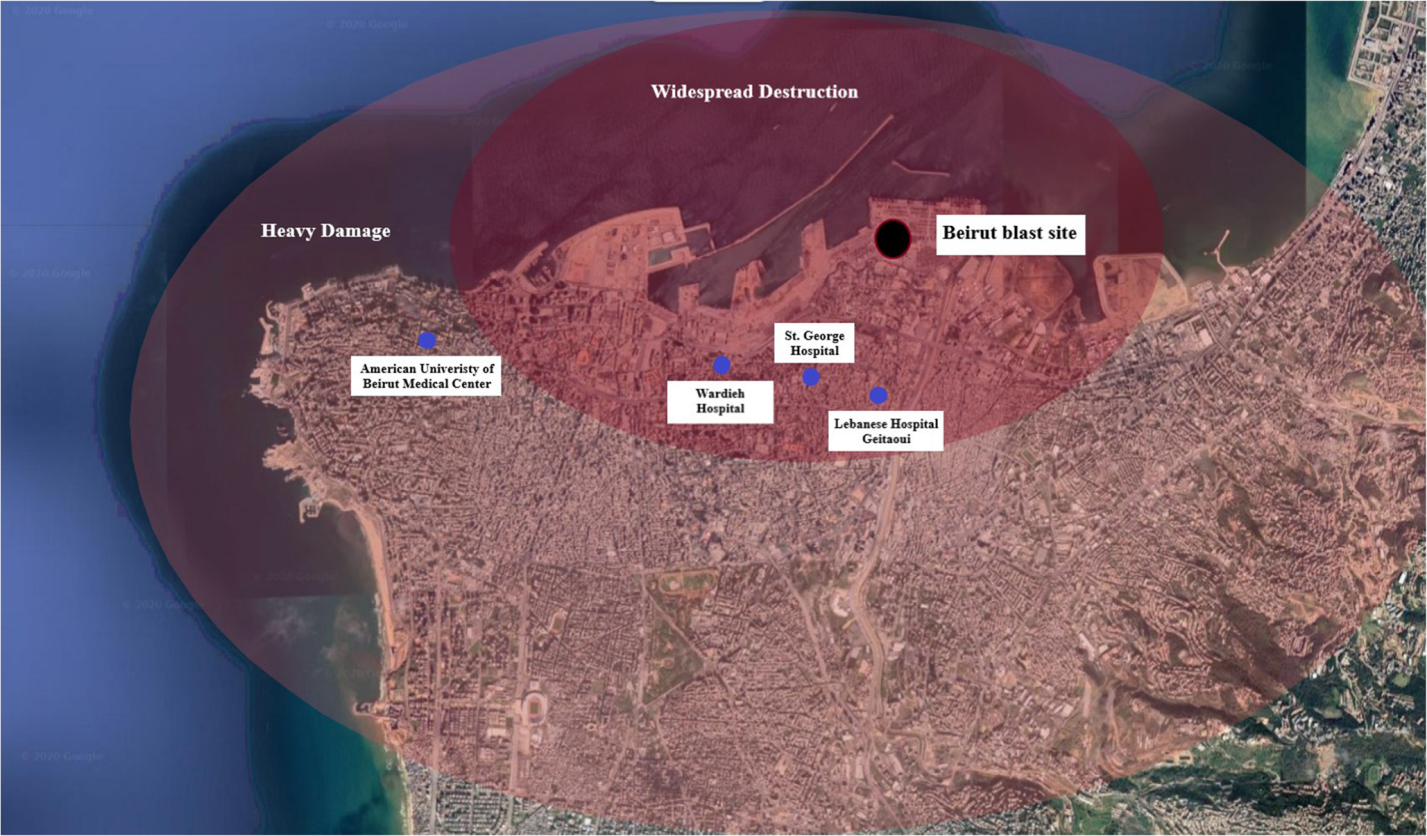
Impact of the Beirut blast on the surrounding area and nearby hospitals

On August 4, 2020, a harrowing blast was heard all over the country, wreaking havoc to the capital and its inhabitants. The blast radius affected a huge portion of the city and caused substantial damage to the health sector; at least one hospital was completely out of function, and three others were significantly damaged and disrupted (Fig. 4) (Dyer 2020). To exacerbate the already challenging healthcare situation, within minutes, the remaining hospitals in the capital were flooded with thousands of cases affected by the blast (Dyer 2020). Emergency protocols were implemented, volunteering medical staff and personnel flooded to the scene, blood banks were in desperate need of donors, and patients were treated in below par conditions (Dyer 2020). The urgent crowded nature of the disaster sidelined COVID-19 infection prevention and control (IPC) protocols, caused mass gatherings around hospitals, and shifted resources dedicated to COVID-19 toward dealing with the Beirut blast casualties.

In addition, and following the explosion, the city’s population took to the streets, and mass gatherings took place (Devi 2020; Oxford Analytica 2020a; Oxford Analytica 2020b). The ammonium nitrate stores were stored improperly next to fireworks and other flammable materials, and the details behind their presence in the port remained ambiguous (Devi 2020). As a result, massive demonstrations were held to protest the government’s incompetence and negligence, and this led to the government’s resignation (Oxford Analytica 2020a; Oxford Analytica 2020b; Oxford Analytica 2020c). Moreover, many volunteers rushed to help clean up the city, conduct minor rehabilitation of households, and prepare and distribute meals for the homeless (Devi 2020; Homsi 2020). Concurrently, thousands of displaced families were in desperate need for proper shelter and housing, and many were forced to reside in crowded unsafe settings (Devi 2020; Homsi 2020). All these circumstances led to an increase in crowding incidents with minimal or no IPC precautions applied.

### Aftermath and impact on the COVID-19 situation

These factors were understandably associated with a spike in COVID-19 cases in Lebanon. The daily number of positive cases following the blast, along with the number of deaths, increased prominently. Incidence rates started increasing significantly approximately a week following the blast, which makes sense given that the virus has an incubation period of 4–7 days (Li et al. 2020). To delineate this effect, the positivity rate on August 4, 2020—date of the explosion—was 2.7 per 100 tests. One week following the Beirut blast, that rate almost doubled to 5.2 per 100 tests, and continued rising till it reached a peak of 10.5 per 100 tests on August 22, 2020, 18 days following the explosion. This rapid increase caused the resigned government to implement a 2-week shutdown as of late August to help prevent spread of the disease (Lebanon 2020). The shutdown involved a public curfew and enforced the temporary closure of restaurants and businesses; the airport was not closed due to the need of influx of foreign currency (Lebanon 2020). Some demographic features of the spread were also altered, as the portion of expat patients decreased following the Beirut blast, probably due to the rise of local cases, and the dissuasion of further expats from returning to the country.

The Beirut blast posed many challenges to the already struggling healthcare sector in Lebanon (Farha and Abi Jaoude 2020). The country was already suffering from the limited medical resources, protective equipment and hospital beds available for COVID-19. The blast further exacerbated the situation by inflicting significant damage to a number of nearby hospitals in the city, overflooding the remaining hospitals with cases, displacing thousands of families without proper care or shelter, and shifting the resources toward dealing with injured individuals (Devi 2020; Dyer 2020; Lebanon 2020). In addition, the mass gatherings that followed the blast did not adhere to IPC measures, and hence, increased the risk of further spread (Oxford Analytica 2020a; Oxford Analytica 2020b). Amid the horrific tragedy of the Beirut blast, an underlying healthcare disaster was slowly growing. Effective measures needed to be implemented swiftly in order to help prevent further spread of the disease. Strict quarantine measures were set, foreign aid was requested, and additional hospitals were recruited to help increase the medical resources available to combat the pandemic, amidst this horrifying tragedy. 

### Limitations

To our knowledge, this is the first Lebanese study to address, both quantitively and qualitatively, the impact of the Beirut blast on the COVID-19 situation in the country. Nevertheless, a few limitations exist. Our data were limited by the retrospective nature of this study, and thus, additional variables and associations could not have been explored. Moreover, we relied on the MOPH website to extrapolate the number of COVID-19 patients in the country; this number could have been under-reported due to the stigmatization of the disease, and hesitancy to contact government officials.

## Conclusion

The Beirut blast hit the city on August 4, 2020, causing immense challenges with respect to the health sector and the current COVID-19 situation in Lebanon. The crises-stricken country was already struggling with the limited medical resources attained for combatting the virus, and the explosion only managed to exacerbate the situation. Hospitals were wrecked, medical resources were consumed, hospital beds were filled with casualties, and preventive measures were ignored.

Aside from the psychologic and socioeconomic impact of the blast, its effects on the COVID-19 pandemic were detrimental. Desperate measures are needed to help the health sector regain its ground, and these include financial reimbursement, foreign aid requests, strict public measures, and recruitment of additional hospitals for managing COVID-19 patients.

## Data Availability

This is a descriptive epidemiological study where the data is deidentified and is publicly accessible on the LMOPH website.\.
